# Investigating the Role of Empagliflozin in the Pathological Progress of Ischemia-Reperfusion Injury in Rat Kidneys: The Involvement of Nitric Oxide

**DOI:** 10.34172/apb.025.46028

**Published:** 2025-10-19

**Authors:** Amin Hasanvand, Abdolreza Tajdar, Azita Zafar Mohtashami, Zahra Haghighatian, Elham Goodarzi, Peyman Amanolahi Baharvand, Babak Hadian

**Affiliations:** ^1^Department of Physiology and Pharmacology, School of Medicine, Lorestan University of Medical Sciences, Khorramabad, Iran; ^2^Student Research Committee, Lorestan University of Medical Sciences, Khorramabad, Iran; ^3^Department of Nephrology, Faculty of Medicine, Lorestan University of Medical Sciences, Khorramabad, Iran; ^4^Department of Pathology, School of Medicine, Lorestan University of Medical Science, Khorramabad, Iran; ^5^Department of Biostatistics and Epidemiology, School of Health and Nutrition, Lorestan University of Medical Sciences, Khorramabad, Iran; ^6^Department of English, School of Medicine, AJA University of Medical Sciences, Tehran, Iran

**Keywords:** Renal, Ischemia-reperfusion, Empagliflozin, Nitric oxide, Rat

## Abstract

**Purpose:**

Renal ischemia-reperfusion (RIR) is a pathological condition that can lead to severe outcomes due to damage to kidney structures. Additionally, oxidative stress is triggered by mitochondrial disruption during reperfusion, potentially resulting in necrosis and, ultimately, cell death through the destruction of cellular membranes.

**Methods:**

Thirty rats were included in this study and divided into the following groups: healthy rats, an ischemia-reperfusion (I/R) group, an I/R group treated with empagliflozin, an I/R group treated with empagliflozin plus L-NAME, and an I/R group treated with empagliflozin plus L-arginine. The drugs were administered from three days before I/R induction to one day post-operation. Blood samples were collected 24 hours after I/R induction to evaluate renal function, inflammatory markers, and oxidative stress. Subsequently, the right kidney was harvested for nitric oxide (NO) measurement, while the left kidney was used for histological analysis.

**Results:**

Empagliflozin administration significantly reduced creatinine, urea, inflammatory markers, and oxidative stress levels. Moreover, empagliflozin increased the levels of antioxidant enzymes and NO. Histopathological analysis indicated that empagliflozin mitigated ischemia-reperfusion injury in renal tissue. The protective effects were further enhanced with the co-administration of empagliflozin and L-arginine. In contrast, simultaneous treatment with empagliflozin and L-NAME led to pathological changes associated with ischemia-reperfusion and attenuated the beneficial effects of empagliflozin.

**Conclusion:**

The findings of this study suggest that empagliflozin exerts protective effects against ischemia-reperfusion injury, likely through the NO pathway.

## Introduction

 Renal ischemia-reperfusion (RIR) is a critical pathological condition that causes structural and functional damage to the kidneys.^1^ During renal ischemia, anaerobic conditions reduce ATP production, leading to cell membrane damage, cellular swelling, and activation of inflammatory pathways.^2^ Renal reperfusion elevates oxidative stress, leading to mitochondrial dysfunction, cell membrane damage, and necrotic cell death.^2^ Numerous studies indicate that inflammatory cytokines and oxidative stress during reperfusion significantly contribute to renal injury and tubular damage.^3,4^ Renal proximal tubular epithelial cells are highly susceptible to ischemia, and hypoxia can compromise nephron structural integrity.^5^ During renal reperfusion, the S3 segment of proximal tubules undergoes apoptosis and necrosis, potentially resulting in renal dysfunction.^6^ Nitric oxide is one of the most important vasodilator enzymes and plays a central role in the regulation of renal hemodynamics.^7^ Reduced nitric oxide (NO) production in the kidneys may contribute to the progression of chronic kidney disease.^8,9^ Studies have also shown that kidney tissue nitrite levels are significantly reduced during ischemia-reperfusion.^10,11^ Studies show that NO protects the kidneys by enhancing ATP synthesis, inhibiting apoptosis, and reducing inflammatory cytokine activity.^12^ It may also lower the risk of acute kidney injury in patients undergoing cardiopulmonary bypass surgery.^13^ Empagliflozin, a sodium-glucose cotransporter-2 (SGLT2) inhibitor, promotes renal glucose excretion and demonstrates renoprotective effects in various models.^14^ Chang Chu et al. found that empagliflozin reduced kidney injury molecule-1 expression and restored normal miR-26a levels in an ischemia-reperfusion model.^15^ Additionally, empagliflozin can reduce intraglomerular pressure, lower the risk of end-stage renal disease, improve kidney filtration, and enhance renal function.^16,17^ It exerts protective effects by increasing GSK-3β phosphorylation during renal ischemia-reperfusion and by activating the AMPK–OPA1 signaling pathway.^18,19^ Several studies have also shown that empagliflozin increases NO activity and bioavailability, contributing to its renal protective effects.^20,21^ Given the role of NO in kidney protection and its stimulation by empagliflozin, we evaluated empagliflozin’s effects on the NO signaling pathway in this study. We further assessed its impact on renal structure, tissue nitrite levels, inflammatory cytokines, and oxidative stress during renal ischemia-reperfusion.

## Materials and Methods

###  Animals and group descriptions

 In this experimental study, thirty male Wistar rats weighing 160–180 g were used. During the study, the animals were maintained under controlled conditions at 21–25 °C with a 12-hour light/dark cycle and housed in standard polyethylene cages. Empagliflozin (Actoverco, Tehran, Iran), L-arginine (Merck KGaA, Darmstadt, Germany), and L-NAME (Sigma-Aldrich, St. Louis, MO, USA) were used. After a two-week adaptation period to laboratory conditions, the animals were randomly allocated into five groups: Group 1, healthy rats without ischemia-reperfusion (the sham group); Group 2, ischemia-reperfusion rats (the vehicle group); Group 3, ischemia-reperfusion rats treated with empagliflozin (10 mg/kg, gavage); Group 4, ischemia-reperfusion rats treated with empagliflozin (10 mg/kg, gavage) and L-NAME (20 mg/kg, intraperitoneally); and Group 5, ischemia-reperfusion rats treated with empagliflozin (10 mg/kg, gavage) and L-arginine (200 mg/kg, intraperitoneally). The doses of empagliflozin, L-arginine, and L-NAME were selected based on previously published studies.^22-25^

###  Ischemia reperfusion induction

 Three days before reperfusion ischemia induction, the drugs were administered daily until the day after surgery. To induce ischemia-reperfusion, the rats underwent a 24-hour fasting period followed by anesthesia with pentobarbital. Both kidneys were subjected to ischemia-reperfusion surgery according to the established protocol. After anesthesia, the lateral areas of the rats were shaved and disinfected. A surgical incision was made to expose the kidneys, and the renal artery was identified. Blood flow was then occluded for 45 minutes using a vascular clip to induce ischemia. Reperfusion was initiated by removing the clip, and the surgical site was sutured. The day after ischemia-reperfusion induction, the rats were re-anesthetized for cardiac blood collection. Both kidneys were harvested for pathological examination and NO measurement. Finally, the animals were euthanized with ether according to established guidelines.^26^

###  Assessment of kidney biomarkers

 After collecting blood from the heart, the samples were centrifuged to separate the serum. Serum creatinine and urea levels were then measured using commercial laboratory kits (Pars Azmoun, Iran) following the manufacturer’s instructions. These measurements were performed to assess renal function in the experimental animals.^27^

###  Assessment of oxidative stress and inflammatory enzymes levels

 Blood samples collected from the heart were centrifuged at 3,500 rpm for 20 minutes to separate the serum. The supernatant was then used to measure oxidative stress markers, including malondialdehyde (MDA) and catalase (CAT), as well as inflammatory enzymes, such as TNF-alpha and CRP. All measurements were performed using commercially available ELISA kits (Abcam, USA) following the manufacturer’s protocols. These analyses were conducted to evaluate oxidative stress and inflammatory responses in the experimental animals.^28^

###  Assessment of tissue nitrite levels

 The right kidney was carefully excised to determine NO concentration. For this purpose, 0.5 mL of tissue homogenate was mixed with 0.1 mL of sulfosalicylic acid and incubated for 30 minutes. The mixture was then centrifuged at 5,000 rpm for 15 minutes to obtain a protein-free supernatant, which was subsequently used for nitrite estimation. A volume of 30 μL of 10% NaOH was added to 200 μL of the supernatant, followed by 300 μL of Tris-HCl buffer, and the solution was thoroughly mixed. Thereafter, 530 μL of Griess reagent was added, and the mixture was incubated in the dark for 10–15 minutes. Absorbance was measured at 540 nm against a Griess reagent blank. Sodium nitrite solution was employed as the standard, and nitrite concentrations in the samples were calculated using standard calibration curves.^29^

###  Histopathology

 The left kidney was carefully excised for histopathological examination. Samples were fixed in 10% buffered formalin and processed using a tissue processor. Sequential procedures, including tissue dehydration, embedding, sectioning, and hematoxylin-eosin staining, were performed in the pathology laboratory. To ensure blinding, all slides were numerically coded prior to assessment. Histological evaluation was conducted by a blinded pathologist according to predefined criteria for inflammation, tubular injury, and glomerular alterations. The kidney samples were specifically examined for inflammation, renal tubular dilation, glomerular mesangial proliferation, hemorrhage, and tubular degeneration.^30^ Histopathological images were acquired using Leica microscope imaging software.

###  Statistical analysis

 Data are presented as mean ± standard deviation (SD) and analyzed using PRISM software. Before performing parametric analyses, normality and homogeneity of variances were assessed using the Shapiro–Wilk and Levene’s tests, respectively. One-way ANOVA was then applied, followed by Tukey’s post-hoc test for multiple comparisons. A *P* value of less than 0.05 was considered statistically significant.

## Results

###  Evaluation of creatinine levels

 As shown in Table 1, rats subjected to ischemia-reperfusion exhibited a significant increase in creatinine levels compared with the healthy (sham) group (*P*< 0.001). Administration of empagliflozin alone (group 3) or in combination with L-arginine (group 5) significantly reduced creatinine levels compared with the ischemia-reperfusion vehicle group (group 2) (*P* < 0.001). Group 4, which received empagliflozin with L-NAME, also showed a reduction in creatinine levels compared with group 2, but this difference was less pronounced (*P* < 0.05).

**Table 1:** Serum creatinine and urea levels in different experimental groups following ischemia-reperfusion (I/R) injury and treatment with empagliflozin and nitric oxide modulators

**Biochemical parameters**	**Groups**				
	**Healthy Animals**	**I/RGroup**	**I/R Group+Empagliflozin**	**I/R Group+L-NAME**	**I/R Group+L-Arginine**
Creatinine (mg/dL)	0.57	1.51^&&&^	1.03^&&&/***^	1.19^&&&/*^	0.87^&/***/@@^
Urea (mg/dL)	34.69	79.42^&&&^	65.71^&&&/*^	72.67^&&&^	49.52^&&/***/$$/@@@^

Data represent the mean values from six animals per group. Statistical analysis was performed using one-way ANOVA followed by Tukey’s post-hoc test. ^*^P < 0.05, ^**^P < 0.01, ^***^P < 0.001 compared with the ischemia/reperfusion group. ^$^*P* < 0.05, ^$$^*P* < 0.01, ^$$$^*P* < 0.001 compared with the empagliflozin group. ^@^*P* < 0.05, ^@@^*P* < 0.01, ^@@@^*P* < 0.001 compared with the group receiving empagliflozin and L.NAME. IR significantly increased serum creatinine and urea levels compared with healthy controls. Treatment with empagliflozin reduced both parameters, indicating renal protection. Co-administration of empagliflozin with L-arginine further improved renal function, showing the lowest creatinine and urea values. In contrast, co-administration with L-NAME (a nitric oxide synthase inhibitor) attenuated the protective effects of empagliflozin.

###  Evaluation of urea levels

 As shown in Table 1, rats subjected to ischemia-reperfusion exhibited significantly higher urea levels compared with the healthy (sham) group (*P* < 0.001). Administration of empagliflozin alone (group 3, P < 0.05) or in combination with L-arginine (group 5, *P* < 0.001) significantly reduced urea levels compared with the ischemia-reperfusion vehicle group (group 2). No significant change was observed in group 4 (empagliflozin + L-NAME) compared with group 2. Notably, urea levels in group 5 were significantly lower than in group 3 (*P* < 0.01), whereas groups receiving L-NAME did not differ significantly from group 3. Finally, a significant difference was observed between the L-NAME and L-arginine groups (*P* < 0.001).

###  Evaluation of TNF-α and CRP enzyme levels

**Figure 1:**
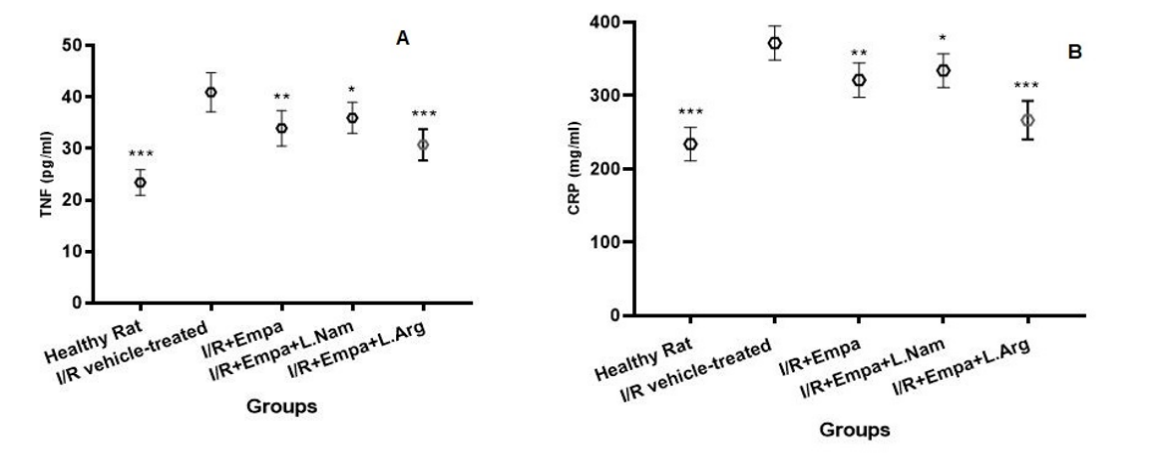
Tumor necrosis factor-alpha (TNF-α, panel A) and C-reactive protein (CRP, panel B) levels in serum following ischemia-reperfusion injury and treatment with empagliflozin and nitric oxide modulators. Cytokine levels were measured in six rats per group. Data are expressed as mean ± SD. Statistical analysis was performed using one-way ANOVA followed by Tukey’s post-hoc test. *P < 0.05, **P < 0.01, ***P < 0.001 vs. IR vehicle-treated group. IR significantly increased TNF-α and CRP levels compared with healthy controls. Empagliflozin treatment reduced both TNF-α and CRP concentrations. Co-administration of empagliflozin with L-arginine enhanced these protective effects, whereas co-administration with L-NAME partially counteracted them. Abbreviations: ischemia-reperfusion, I/R; Empa, empagliflozin; L.Nam, L-NAME; L.Arg, L-arginine

 As shown in Figures 1A and 1B, inflammatory cytokine levels were significantly elevated in the ischemia-reperfusion vehicle group (group 2) compared with the healthy (sham) group (group 1). Administration of empagliflozin (group 3) significantly reduced these cytokine levels (*P* < 0.001). In group 4, inhibition of NO synthesis led to a moderate but significant decrease in inflammatory cytokines compared with group 2 (*P* < 0.05). Notably, stimulation of the NO pathway further enhanced the reduction of inflammatory cytokines in group 5 (*P*< 0.001).

###  Evaluation of catalase and MDA enzyme levels

**Figure 2:**
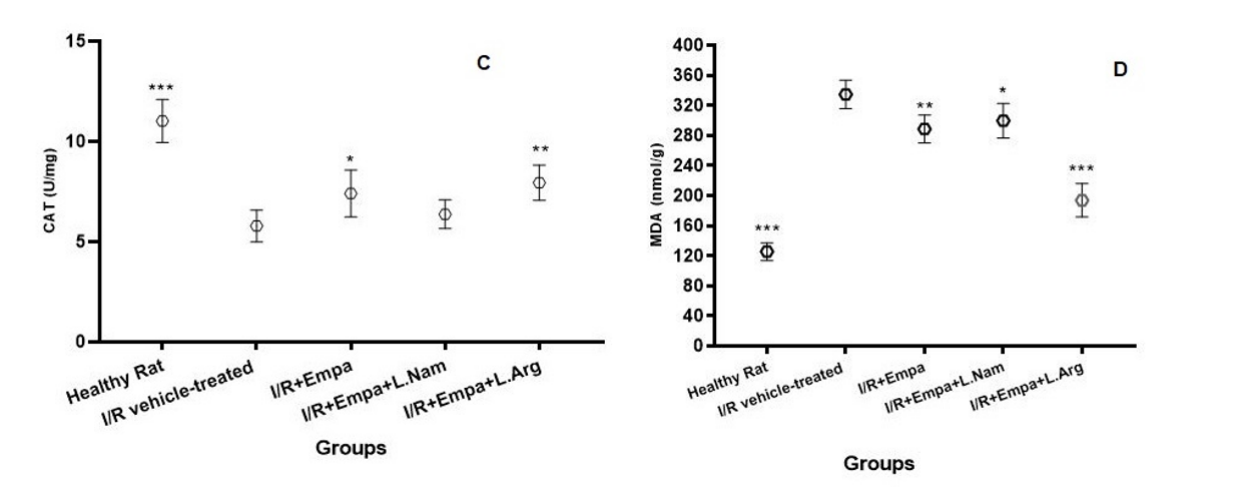
Catalase (CAT, panel C) and malondialdehyde (MDA, panel D) levels in kidney tissue following ischemia-reperfusion injury and treatment with empagliflozin and nitric oxide modulators. Enzyme levels were measured in kidneys of six rats per group. Data are presented as mean ± SD. Statistical analysis was performed using one-way ANOVA followed by Tukey’s post-hoc test. *P < 0.05, **P < 0.01, ***P < 0.001 vs. IR vehicle-treated group. IR significantly decreased CAT activity and increased MDA levels compared with healthy controls. Treatment with empagliflozin partially restored CAT activity and reduced MDA levels. Co-administration of empagliflozin with L-arginine further improved these effects, while co-administration with L-NAME attenuated the protective effects of empagliflozin. Abbreviations: ischemia-reperfusion, I/R; Empa, empagliflozin; L.Nam, L-NAME; L.Arg, L-arginine

 As shown in Figure 2, ischemia-reperfusion induction in the vehicle group (group 2) resulted in a decrease in catalase (CAT) activity and an increase in malondialdehyde (MDA) levels. Administration of empagliflozin (group 3) significantly increased CAT activity (*P* < 0.05) and reduced MDA levels (*P* < 0.01). Co-administration of empagliflozin with L-NAME (group 4) did not significantly affect CAT activity compared with group 2, although MDA levels were moderately reduced (*P* < 0.05). Notably, simultaneous administration of empagliflozin and L-arginine (group 5) significantly increased CAT activity (*P* < 0.01) and markedly decreased MDA levels (*P* < 0.001).

###  Evaluation of Tissue Nitrite Levels

**Figure 3:**
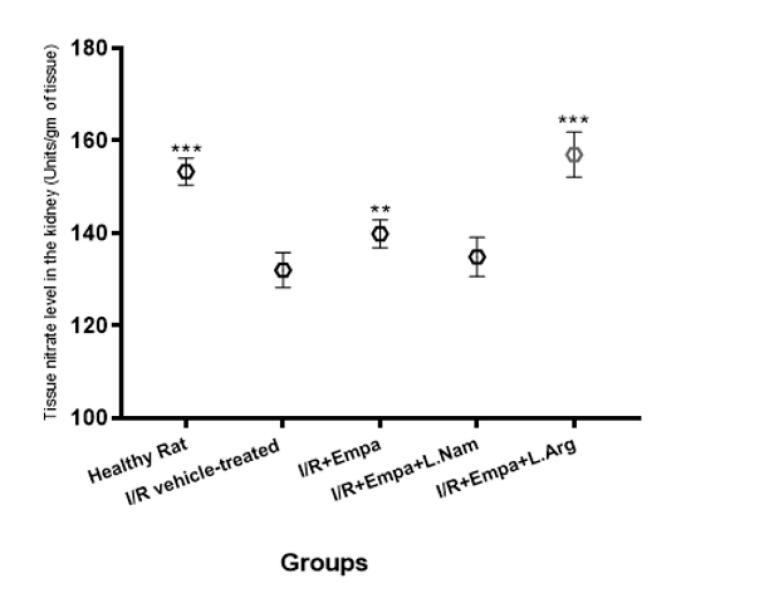
Tissue nitrite levels in the kidney following ischemia-reperfusion injury and treatment with empagliflozin and nitric oxide modulators. Tissue nitrite levels were measured in kidneys of six rats per group. Data are expressed as mean ± SD. Statistical analysis was performed using one-way ANOVA followed by Tukey’s post-hoc test. *P < 0.05, **P< 0.01, ***P < 0.001 vs. IR vehicle-treated group. IR significantly reduced tissue nitrite levels compared with healthy controls. Empagliflozin treatment alone or in combination with L-arginine significantly increased nitrite levels, while co-administration with L-NAME did not significantly alter nitrite levels compared with the IR group. Abbreviations: ischemia-reperfusion, I/R; Empa, empagliflozin; L.Nam, L-NAME; L.Arg, L-arginine

 As shown in Figure 3, tissue nitrate levels in the kidneys were significantly reduced in the ischemia-reperfusion vehicle group (group 2) compared with the healthy (sham) group. Administration of empagliflozin (group 3) significantly increased NO levels (*P* < 0.01), and co-administration of empagliflozin with L-arginine (group 5) further enhanced this effect (*P* < 0.001). In contrast, co-administration of empagliflozin with L-NAME (group 4) resulted in decreased NO levels, which were not significantly different from those in group 2.

###  Histopathological Evaluation

**Figure 4:**
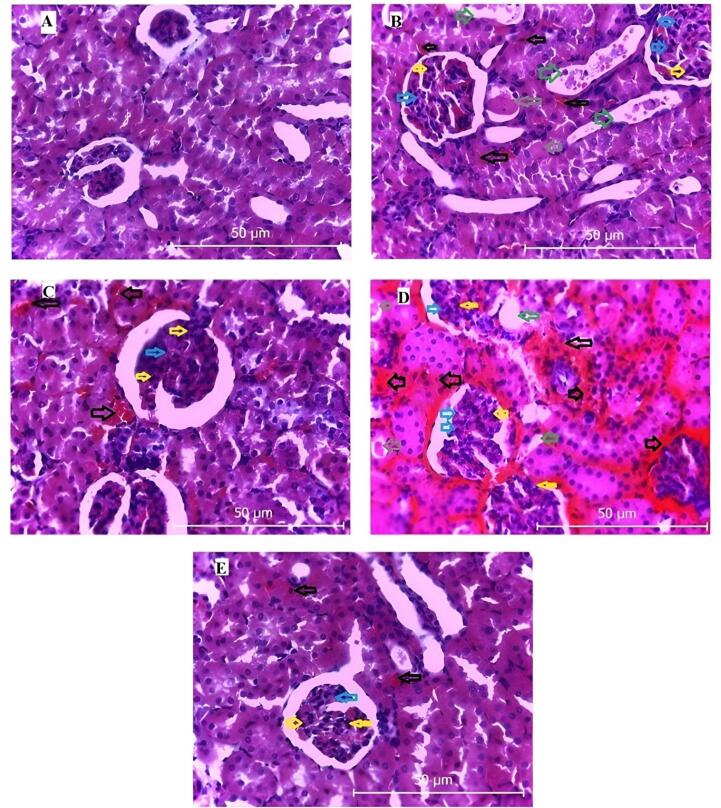
Renal histopathology following ischemia-reperfusion (I/R) and treatment with empagliflozin and nitric oxide modulators (H&E, × 400). (A) Control group with normal renal structure. (B) I/R group showing severe injury, including inflammation (yellow arrows), tubular dilation (green arrows), mesangial proliferation (blue arrows), hemorrhage (black arrows), and tubular degeneration (gray arrows). (C) I/R + empagliflozin: partial recovery with reduced tissue lesions. (D) I/R + empagliflozin + L-NAME: attenuated protection with persistent pathological changes. (E) I/R + empagliflozin + L-arginine: marked improvement with decreased inflammatory and degenerative alterations

 Hematoxylin and eosin-stained kidney sections were examined, and pathological changes were scored on a semi-quantitative scale (0–4) for inflammation, tubular injury, hemorrhage, and glomerular mesenchymal proliferation. In the ischemia-reperfusion (RIR) group (Figure 4B), kidney tissues exhibited pronounced pathological alterations, with high scores for inflammation (3.5 ± 0.3), hemorrhage (3.0 ± 0.4), focal tubular injury (3.5 ± 0.2), tubular dilation and degeneration (3.0 ± 0.3), and glomerular mesenchymal proliferation (3.0 ± 0.2). Empagliflozin treatment (group 3, Figure 4C) significantly reduced these scores, indicating attenuated inflammation and tissue injury. Conversely, co-administration of empagliflozin with L-NAME (group 4, Figure 4D) resulted in worsened pathological scores, reflecting increased inflammation, hemorrhage, tubular damage, and glomerular mesenchymal proliferation. Combined treatment with empagliflozin and L-arginine (group 5, Figure 4E) significantly improved histological scores, demonstrating marked alleviation of inflammation, tubular injury, hemorrhage, and glomerular mesenchymal proliferation compared with the RIR group.

## Discussion

 The findings of the present study demonstrate that RIR induces significant oxidative stress and inflammatory responses, which contribute to structural kidney damage. Histological analysis revealed prominent pathological alterations, including hemorrhage, tubular degeneration and dilation, inflammation, and glomerular mesenchymal proliferation. These structural changes were accompanied by elevated serum levels of urea and creatinine, reflecting impaired renal function. Treatment with empagliflozin markedly attenuated these pathological changes, reduced oxidative stress, and decreased the levels of inflammatory cytokines, thereby improving renal function. These results are consistent with previous reports highlighting the renoprotective and anti-inflammatory effects of SGLT2 inhibitors in ischemic kidney injury. Interestingly, co-administration of L-NAME, a nitric oxide synthase inhibitor, with empagliflozin attenuated the drug’s protective effects. This combination led to increased oxidative stress and inflammatory markers, as well as worsened histopathological indicators, suggesting that the therapeutic benefits of empagliflozin are at least partly mediated via the NO pathway. Conversely, simultaneous administration of empagliflozin with L-arginine, a nitric oxide precursor, further enhanced renoprotection by significantly reducing oxidative stress and inflammatory cytokines and improving histological indices beyond those observed with empagliflozin alone. Collectively, these results indicate that empagliflozin exerts protective effects against ischemia-reperfusion-induced renal injury primarily through modulation of the NO signaling pathway. The combination of empagliflozin with NO stimulation may offer a synergistic approach to mitigate oxidative stress, inflammation, and structural kidney damage, thereby improving functional outcomes following renal ischemia-reperfusion.

 The results of this study demonstrate that inhibition of the NO pathway by L-NAME markedly reduces the protective effects of empagliflozin, highlighting the critical role of NO signaling as a therapeutic target in ischemia-reperfusion-induced renal injury. These findings are consistent with previous studies emphasizing the critical role of NO in regulating renal hemodynamics, glomerular function, and inflammatory responses.^31,32^ Furthermore, co-administration of empagliflozin with L-arginine, a nitric oxide precursor, further enhanced renal function and attenuated tissue damage, suggesting a synergistic protective effect. These results underscore the potential of combination therapies in managing kidney injuries and emphasize the critical role of NO homeostasis in maintaining renal physiology. Maintaining renal physiology is crucial, and low-dose sodium nitrite, a nitric oxide donor, has been shown to protect against renal ischemia-reperfusion injury by enhancing renal oxygenation.^33^ Furthermore, this study confirms the renoprotective effects of empagliflozin and emphasizes the critical role of NO signaling pathways in kidney function. The results indicate that ischemia-reperfusion injury reduces NO levels in kidney tissue, which is associated with increased oxidative stress and elevated inflammatory mediators, ultimately causing structural renal damage. These findings align with previous studies reporting decreased NO bioavailability during renal ischemia-reperfusion injury.^34^ In the present study, tissue nitrate measurements in the second group revealed a reduction in NO levels in kidneys subjected to ischemia-reperfusion.

 Moreover, numerous studies have demonstrated the renoprotective effects of empagliflozin. Clinical evidence indicates that, in addition to its safety, empagliflozin effectively reduces the risk of kidney disease progression and associated mortality in patients with chronic kidney disease.^35^ In chronic kidney disease, the loss of functional nephrons increases the workload on remaining nephrons, leading to elevated intraglomerular pressure, hyperfiltration, and progressive structural damage such as glomerulosclerosis and tubulointerstitial fibrosis. These injurious processes are precisely the targets of SGLT2 inhibitors, which mitigate disease progression by reducing glomerular pressure, tubular workload, and inflammation.^36^ Empagliflozin has been shown to increase urinary adenosine excretion and reduce hyperfiltration by promoting afferent arteriolar constriction.^37^ Additionally, glycosylation can trigger afferent glomerular arteriole contraction via tubuloglomerular feedback, leading to decreased glomerular pressure and filtration rate.^36^ Studies also indicate that empagliflozin can lower serum urea and albumin levels and mitigate both tubulointerstitial and glomerular damage.^38^ These findings are consistent with the results of the present study. A key distinction, however, is that our research specifically investigates the role of the NO signaling pathway. Empagliflozin preserves renal tissue structure during ischemia-reperfusion injury by significantly reducing the expression of pro-apoptotic markers such as BAX, Caspase-3, and Caspase-8.^39^ The elevated NO levels observed in empagliflozin-treated groups, especially when combined with L-arginine, indicate activation of the NO pathway, which likely represents a central mechanism underlying its renoprotective effects. This activation may be mediated through enhanced endothelial nitric oxide synthase (eNOS) activity and decreased oxidative stress, as reported in previous studies.^40,41^ Empagliflozin has been shown to enhance the bioavailability of NO by modulating and inhibiting reactive oxygen species, without altering eNOS expression or signaling.^42,43^ In a gentamicin-induced kidney injury model, empagliflozin increased PAX2 levels and reduced inflammatory mediators.^44^ PAX2, a key transcription factor, plays a critical role in kidney tissue repair and limits fibrosis.^45^ Additionally, the observed reductions in inflammatory cytokines and improvements in histological indices in empagliflozin-treated groups highlight its anti-inflammatory and antifibrotic effects, likely mediated through the modulation of inflammation and restoration of oxidative balance, consistent with its reported antioxidant properties.^46,47^ Histopathological analyses have demonstrated that empagliflozin treatment effectively mitigates cystic alterations, inflammation, tissue expansion, and overall renal damage in experimental models of chronic interstitial kidney fibrosis.^48^ Moreover, empagliflozin has been shown to provide renal benefits not only in diabetic patients but also in those with non-diabetic kidney disease.^35,49,50^ Several studies have suggested that NO plays a crucial role in protecting against kidney diseases by reducing oxidative stress and inflammatory mediators.^51,52^ In the present study, empagliflozin treatment improved biochemical and histological parameters in rats, and these effects were further augmented when combined with L-arginine. The observed increase in tissue nitrite levels suggests a potential involvement of NO-related signaling in mediating the renoprotective effects of empagliflozin. Increased NO bioavailability may contribute to reduced oxidative stress and inflammation; however, it should be noted that nitrite was only used as a surrogate marker, and we did not directly assess eNOS activity, phosphorylation status, or NOS isoform expression. Importantly, empagliflozin is known to exert pleiotropic effects beyond glycemic control, including AMPK activation, reduction of oxidative stress, and anti-inflammatory actions, which may also contribute to the observed protective outcomes, either independently or in concert with NO signaling.

## Conclusion

 The results of this study demonstrated that empagliflozin administration significantly protects against kidney damage induced by ischemia-reperfusion by reducing inflammatory cytokines and oxidative stress. This renoprotective effect of empagliflozin was markedly attenuated when co-administered with L-NAME, whereas it was enhanced with L-arginine co-administration. The observed reduction in pathological damage—including inflammation, tubular dilation, glomerular mesenchymal proliferation, hemorrhage, and tubular degeneration—suggests that the NO pathway represents a key mechanism underlying the renal protective effects of empagliflozin. The researchers recommend that future studies investigate levels of PAX2 and other relevant markers, which were not examined in this study due to its limitations. These findings provide a promising basis for the development of targeted therapies aimed at preventing or mitigating ischemia-reperfusion injury in the kidneys and underscore the need for further research into these molecular pathways and their complex interactions.

## Limitations and Future Directions

 Absence of direct molecular validation of NO-related pathways, such as eNOS phosphorylation or NOS isoform expression, was a limitation for this study. Due to resource constraints, we focused primarily on functional and biochemical indices (renal function markers, oxidative stress parameters, and tissue nitrite levels), which are well-established surrogate indicators of NO bioavailability in ischemia-reperfusion injury models. In addition, although the combination of empagliflozin and L-arginine demonstrated enhanced protective effects, we did not conduct a formal synergy analysis, and thus the observed outcomes should be regarded as additive rather than definitively synergistic. Another limitation is the relatively small sample size (n = 6 per group), which may reduce statistical power and generalizability. Future studies with larger cohorts and incorporation of molecular assays (e.g., western blotting, qPCR) are warranted to validate NO pathway activation and to further elucidate the mechanistic basis of empagliflozin’s renoprotective effects.

## Competing Interests

 The authors have no conflict of interest to declare.

## Data Availability Statement

 The datasets used and/or analyzed during the present study are available from the corresponding author on reasonable request.

## Ethical Approval

 This study was approved by the Ethics Committee of Lorestan University of Medical Sciences with the code IR.LUMS.REC.1401.256.
